# Drug-drug co-crystals

**DOI:** 10.1186/2008-2231-20-45

**Published:** 2012-10-04

**Authors:** Bhupinder Singh Sekhon

**Affiliations:** 1 PCTE Institute of Pharmacy, near Baddowal Cantt, Ludhiana, 142 021, India

## Editorial

**Figure 1:**
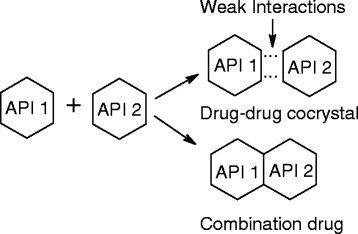
Representation of drug-drug co-crystal and combination drug.

Active pharmaceutical ingredients (APIs) are most conveniently developed and delivered orally as solid dosage forms that contain a defined crystalline form of an API. Co-crystal is a crystalline entity formed by two different or more molecular entities where the intermolecular interactions are weak forces like hydrogen bonding and π-π stacking. Co-crystals are an enabling technology that is used in new or existing drug delivery systems by majority of pharmaceutical companies in formulation and drug development. The concept of modifying the properties of a drug molecule by forming a pharmaceutical co-crystal containing a single APIs and a pharmaceutical relevant co-former with improved properties compared with the pure drug crystal has generated immense interest [1]. Physicians prescribe combination therapy frequently to treat and manage a plethora of medical conditions. Multi-API co-crystals, relatively unexplored solid forms of APIs, have potential relevance in the context of combination drugs for pharmaceutical drug development (Figure 1).

The idea of developing multi-API co-crystals is interesting. This is reflected from the number of publications and patent applications for co-crystals in recent years. Drug-drug co-crystals fulfil the criteria for patent eligibility: novelty, utility, and non-obviousness for pharmaceutical development. However, no compilation of drug-drug co-crystals information’s is available in literature***.*** There is immense potential to explore co-crystal design of established APIs among each other to enhance solubility and bioavailability of the product. Consequently, there is a strong need to devise ways to increase the likelihood of success in generating drug–drug co-crystals. In this context, the limited available reports in literature are described here. While co-administering a combination of theophylline and phenobarbital, it was discovered that a co-crystal of 2:1 stoichiometry existed between the two compounds [2]. The example of a trimorphic co-crystal involving APIs i.e. ethenzamide and gentisic acid, may find relevance in the context of combination drugs [3]. The meloxicam-aspirin co-crystal decreased the time required to reach the human therapeutic concentration compared with the parent drug, meloxicam [4]. Higher degree of solubility of acetylsalicylic acid: (*L*)-theanine [(*L*)-5-*N*-ethyl-glutamine] co-crystal system enables aspirin formulations for intravenous administration [5]. The 1:1 acetaminophen/theophylline (AT) co-crystal had a faster dissolution rate than AT physical mixtures [6]. Pharmaceutical composition comprising therapeutically effective amount of lamivudine: zidovudine co-crystals use for manufacture of medicament for treatment of HIV infections of humans was reported [7]. A single step co-deposition of albuterol sulphate: ipratropium bromide co-crystal aerosols was reported [8]. Two 1:1 drug-drug co-crystals [isoniazid: 4-aminosalicylic acid; pyrazinamide: 4-aminosalicylic acid] may be exploited for the treatment of tuberculosis [9]. Celecoxib: venlafaxine co-crystal and tramadol: celecoxib co-crystals use as medicaments, more particularly for the treatment of pain has been reported [10]. Amoxicillin-clavulanate co-crystal improved its antibiotic activity against non-beta lactamase bacterial, *Sarcina lutea*[11]. Further, many pharmaceutical companies and various groups are working actively on co-crystals. Success has been achieved for various co-crystal systems such as sulfamethazine-theophylline, pyrazinamide-diflunisal, sulfamethazine-potassium salt of 4-aminobenzoic acid, theophylline: gentisic acid, ethenzamide-gentisic acid, succinic acid (SA)-pyrazinamide (PZA)–isoniazid (INH) two-drug combination composed of the co-crystals PZA–(SA)_0.5_ and INH– SA_(0.5)_.

In view of the reported generation of pharmaceutical co-crystals containing two active pharmaceutical ingredients mentioned above, drugs having similar structure and similar 3-D arrangement should be exploited for drug-drug synergism to obtain multiple API co-crystals. Co-crystal screening technology has the potential to identify and establish new IP for new drug-drug co-crystals of multiple APIs to protect the product from competition. Further, it offers immense potential in various fields such as resolution of racemic drugs to enantiomerically pure isomers by single pure enantioner of API through co-crystallization. In this way, optimization of co-crystal screening may lead to commercialization of new co-crystal product along with separated single enantiomers. In the case of commercial API-API combination, a patent of a drug-drug co-crystal with better drug properties than previously known forms could be of high commercial value. Designing drug-drug co-crystals of marketed drugs may shorten development period (including clinical trials) than those of New Chemical Entities as co-crystals do not involve structural modification of the APIs.

Co-crystals are less prone to suffer polymorphic transformations and the status of polymorphism in this class of co-crystals needs investigation. Co-crystals among APIs such as aspirin, caffeine, theophylline, sulphadimidine, carbamazepine, fluoxetine hydrochloride, piroxicam, norfloxacin, indomethacin, ibuprofen, paracetamol, flurbiprofen, itraconazole are of interest for multi-API co-crystals study. A single-step, scalable, solvent-free, continuous co-crystallization and agglomeration technology developed for co-crystal agglomerates of ibuprofen: nicotinamide (1:1 ratio) using Hot Melt Extrusion, offer the flexibility for tailoring the co-crystal purity. The potential of supercritical fluids as new media for the co-crystallization of APIs has been addressed recently and screening for pharmaceutical co-crystals using the supercritical fluid enhanced atomization process might help for production of multi-API co-crystals. Experts are of the opinion that multi-API co-crystals are expected to overcome the problems associated with traditional combination drugs and it is hoped that further research in this area may have some bearing in the treatment of several diseases.
